# Policy Interventions, Development Trends, and Service Innovations of Internet Hospitals in China: Documentary Analysis and Qualitative Interview Study

**DOI:** 10.2196/22330

**Published:** 2021-07-20

**Authors:** Yunfeng Lai, Shengqi Chen, Meng Li, Carolina Oi Lam Ung, Hao Hu

**Affiliations:** 1 State Key Laboratory of Quality Research in Chinese Medicine Institute of Chinese Medical Sciences University of Macau Macau China

**Keywords:** internet hospital, health policy, medical service, public hospital, digital health, China

## Abstract

**Background:**

Internet hospitals have been encouraged by the Chinese government to develop an innovative medical service model that mainly uses new internet-based technologies to increase access to health care and improve the quality and efficiency of health care delivery. However, the academic exploration of the institutional and sectoral development of internet hospitals in China is scarce in the existing literature.

**Objective:**

This study aimed to investigate the policy interventions, development trends, and service innovations of internet hospitals in China. It is expected that the findings from this study will contribute to the further innovation of internet hospitals in China and provide references for the international development of internet hospitals for personalized digital health and patient-centric services.

**Methods:**

This study analyzed official policies related to internet hospitals that were implemented by the government in China since 2005. The data of formally approved internet hospitals were collected from official websites to analyze development trends. In-depth semistructured interviews were conducted with 58 key stakeholders who represented comprehensive viewpoints about the service innovations of internet hospitals between March and November 2019.

**Results:**

In total, 25 policies that promoted the development of internet hospitals in China were identified. These policies encompassed informatization infrastructure construction, medical resource integration, development model design, service model design, and payment model design. Of the 268 internet hospitals that had received an official license from the government, 153 public internet hospitals had been built mainly by medical institutions. Public tertiary hospitals were the main actors in founding internet hospitals that were created to provide services that targeted patients with common diseases or chronic diseases or patients living in remote and rural areas. Promoting convenient access to high-quality medical resources and saving patients’ and their families’ time were the key values of internet hospitals.

**Conclusions:**

The policy interventions strongly promoted the development of internet hospitals in China. Public tertiary hospitals led the development of internet hospitals. However, internet hospitals in China have mainly played roles that are complementary to those of physical medical institutions. The service model of internet hospitals needs more distinguished innovations to provide personalized digital health and patient-centric services.

## Introduction

A total of 10 years have passed since the launch of new health reform in China. Substantial progress in providing equal access to health care and improving financial risk protection has been achieved. However, there are still gaps in medical quality, the control of noncommunicable disease, the control of health expenditures, public satisfaction, and the distribution of medical resources [1]. Indeed, due to the vast population and uneven distribution of medical resources in China, the use of medical resources is contentious [2]. In order to promote China’s health care, there is a need to further reform the medical payment system, the hospital governance structure, primary health care institutions’ capacity-building methods, and health education and a need to make full use of digital medical technology [3-5]. The expected outcomes are the promotion of the construction of health-oriented medical alliances [6-8], improvements in medical service quality through medical insurance payments, and the acceleration of medical service innovation [9]. As such, internet hospitals have been encouraged in recent years by the Chinese government.

In order to develop internet hospitals effectively, the Chinese government issued various relevant policy interventions in the past 15 years. The government aimed to develop an innovative medical service model that mainly used new internet-based technologies to increase access to health care and improve the quality and efficiency of health care delivery. Notably, technological innovations were used to improve health care use across hospitals in China [10]. As an emerging innovation in China, internet hospitals are expected to improve the availability of medical resources and decrease the costs of distant medical services [11].

Telehealth (or telemedicine) is 1 kind of worldwide medical service model that is similar to the internet hospital model in China [12]. In advanced countries like the European Union, the United States, and Japan, telehealth has been studied and practiced for many years, mainly in fields such as ophthalmology, cardiovascular, and dermatology [13]. However, in terms of service scope and means, the internet hospital model in China is more advanced than the traditional telehealth model. In theory, internet hospitals in China are internet-based medical platforms that medical institutions use to directly provide medical services to patients by using information technology to extend medical resources from hospitals to the internet and carry out web-based medical care and health services that can be received by patients in a home setting [14]. The internet hospital model is a new format that is derived from the “Internet + Health Care” format, which is an extension of the telemedicine and traditional hospital formats. By using the medical resources of physical hospitals and internet technology, internet hospitals provide patients with closed-loop medical services (ie, web-based and offline medical services, including web-based registration, web-based medical consultation, web-based drug supply and support, and web-based payment services), so that patients can use the more convenient medical services of physical hospitals and optimize and match existing medical needs and health care resources [15]. It is believed that the scope of internet hospitals not only encompasses telemedicine but also covers electronic prescriptions, medical insurance, commercial health insurance, health management, hospital operation, and hospital logistics. The internet hospital model is expected to become an innovative medical service model that is different from traditional hospital models [16]. In particular, during the COVID-19 pandemic, internet hospitals in China have become important components of infection prevention and control measures by offering essential medical support to the public despite the strict social distancing requirements, promoting self-care and self-protection, and facilitating epidemiological screening [17,18].

At present, the development of internet hospitals is far from mature and is limited by many factors, such as the scarcity of web-based doctors and the unavailability of medical insurance coverage [19]. Existing literature on internet hospitals mainly focuses on informatization infrastructure construction and development model design [20]. Consequently, while internet hospitals have been encouraged by the Chinese government to optimize health care resource use, the academic exploration of the institutional and sectoral development of internet hospitals in China remains scarce in existing literature. Thus, this study aimed to investigate the policy interventions, development trends, and service innovations of internet hospitals in China. It is expected that the findings from this study will contribute to the further innovation of internet hospitals in China and provide references for the international development of internet hospitals for personalized digital health and patient-centric services.

## Methods

### Research Design

This study had a qualitative research design and collected data via the following three methods: (1) policy file collection and analysis; (2) the documentary analysis of internet hospital approval; and (3) qualitative interviews with key stakeholders.

### Policy File Collection and Analysis

To understand the policy interventions on internet hospital development in China, we collected and analyzed the relevant policy files of internet hospitals. We searched for these files on the websites of relevant government departments, including the websites of the State Council, the National Health Commission, the National Administration of Traditional Chinese Medicine, the National Health Security Administration, and the National Medical Product Administration. These websites were searched for relevant policies and regulations describing “Internet + Health Care” and internet hospitals. We used the following search terms: *Internet hospital*, *Internet + health*, *Internet + healthcare*, *Internet medical consultation*, *telemedicine*, *Internet + medical insurance service*, *Internet drug business*, *healthcare big data*, *health information*, *Healthy China 2030*, *tiered healthcare delivery*, *medical treatment combination* (*yi lian ti*), and *medical alliance* (*yi gong ti*). We also used combinations of these terms. The date of the last search was March 10, 2020.

In terms of the inclusion and exclusion criteria, this study only included formal and legal policy documents from official authorities. These selected policy documents were reviewed based on their objectives and their relevance to supporting internet hospitals by (1) providing a legal framework for practice, (2) identifying a development plan, (3) formulating specific development actions, (4) formulating management actions, and (5) formulating security measures.

### Documentary Analysis of Internet Hospital Approvals

To explore the development trends of internet hospitals in China, we conducted a documentary analysis of the official approvals of internet hospitals. With regard to the data source, the data of formally approved internet hospitals that were published up to April 15, 2020, were directly collected from the official websites of provincial health commissions in China—the competent authorities that approve the establishment of internet hospitals in their provinces. The names, locations, owners, and approval times of internet hospitals were directly searched on and extracted from the official websites of the provincial health commissions by our research term. After collecting all of the data, we conducted descriptive statistical analyses to analyze the number, geographical distribution, approval time, ownership, and founding institutions (medical institution vs company) of established internet hospitals.

### Qualitative Interviews With Key Stakeholders

To investigate key stakeholders’ perceptions about the service innovations of internet hospitals, we used a qualitative interviewing method. Between April and November 2019, semistructured interviews were conducted with a wide range of key stakeholders in the internet hospital sector, including policy makers, not-for-profit professional societies, hospital administrators, doctors, managers of pharmaceutical companies, managers of internet companies, information technology engineers, academic researchers, and financial investors.

Purposive sampling was used to select the interviewees. A total of 58 key stakeholders took part in the interviews, and their characteristics are presented in Table 1. There were 44 males and 14 females. Interviewees were from 10 regions and included 26 stakeholders from Beijing, 16 from Guangdong, 6 from Sichuan, 4 from Shanghai, 2 from Zhejiang, 1 from Chongqing, 1 from Tianjin, 1 from Jiangsu, and 1 from Hubei.

**Table 1:** Characteristics of interviewees (N=58).

Characteristics	Interviewees, n
Policy makers	9
Not-for-profit professional societies	5
Hospital management staffs	8
Doctors	5
Pharmaceutical company managers	12
Internet company managers	9
Information technology engineers	2
Academic researchers	7
Financial investors	1

We approached the interviewees in advance to obtain informed consent. In total, 45 interviews were conducted face-to-face, and 13 interviews were conducted via telephone. The interviewees were asked questions that primarily focused on the policy interventions, development trends, and service innovations of internet hospitals. We recorded the audio of all interviews, which were transcribed verbatim with the interviewees’ consent. The average interview time was about 50 minutes.

Thematic analysis was used for the data analysis of qualitative interviews. First, two researchers conducted the thematic analysis separately. Second, they met with each other to identify similarities and the differences by comparing the analysis results. Third, another two researchers analyzed the differences to conduct a triangular test. Finally, the final results of the qualitative investigations were reviewed by all researchers together.

### Data Availability

The data sets generated for this study are available from the corresponding authors upon reasonable request.

### Ethics Statement

The study design was approved by the ethics committee of the University of Macau (approval number: BSERE20-APP004-ICMS).

## Results

### Policy Interventions of Internet Hospitals in China

#### Summary of Policy Interventions

**Figure 1:**
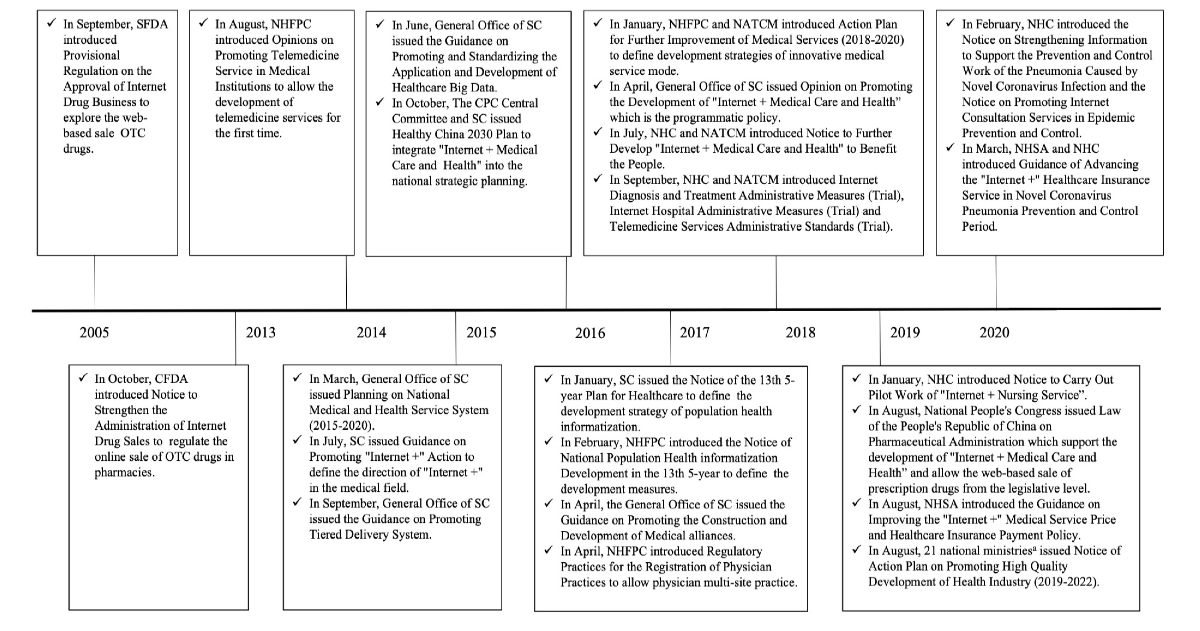
The main policy interventions in the history of internet hospitals in China. These policy interventions were created by the following 21 national ministries: the National Development and Reform Commission, the Ministry of Education, the Ministry of Science and Technology, the Ministry of Industry and Information Technology, the Ministry of Civil Affairs, the Ministry of Finance, the Ministry of Human Resources and Social Security, the Ministry of Natural Resources, the Ministry of Ecology and Environment, the Ministry of Housing and Urban-Rural Development, the Ministry of Commerce, the Ministry of Culture and Tourism, the NHC, The People's Bank of China, State Taxation Administration, the State Administration for Market Regulation, the General Administration of Sport of China, the China Banking and Insurance Regulatory Commission, the National Healthcare Security Administration, the NATCM, and the National Medical Products Administration. CFDA: China Food and Drug Administration; CPC: Communist Party of China; NATCM: National Administration of Traditional Chinese Medicine; NHC: National Health Commission; NHFPC: National Health and Family Planning Commission; NHSA: National Health Security Administration; OTC: over-the-counter; SC: State Council; SFDA: State Food and Drug Administration.

In total, 25 policy files were identified in this study [21-43], including 1 law and 24 regulations. The details of these 25 policy documents are summarized in Multimedia Appendix 1. Corresponding with the official announcement timing of policy interventions, the policy developments of internet hospitals in China can be classified into two phases—phase 1 (2005-2017) and phase 2 (2018 onward; Figure 1).

#### Phase 1 (2005-2017)

In 2005, the State Food and Drug Administration allowed the web-based sales of over-the-counter drugs for the first time, which prompted the exploration of innovative drug services on internet [26]. In 2014, the National Health and Family Planning Commission allowed telemedicine service providers to create medical care services. In 2015, the State Council integrated the “Internet + Health Care” format into the national medical and health service system to promote health information services and smart medical services and benefit the whole population via information technology. Moreover, the State Council issued guidance on building a tiered health care delivery system to reform the existing hospital-centric model by using information technology and big data. In 2016, the country’s long-term health sector strategy—“Health China 2030”—was announced by President XI Jinping. This strategy involved integrating the “Internet + Health Care” format into the national strategic plans for health. Similarly, the use of health care big data in in key informatization infrastructure construction has also been the focus of the State Council. In 2017, the State Council promoted the development of medical institution alliances to operationalize the tiered health care delivery approach, which can strengthen the support and training that higher medical institutions provide to primary medical institutions through digital technology. The establishment of a population health information system was listed as a key project by the State Council and the National Health and Family Planning Commission. Furthermore, the National Health and Family Planning Commission allowed physicians to conduct multisite practices and gave official permission to physicians to work for internet-based medical institutions like internet hospitals.

#### Phase 2 (2018 onward)

In 2018, a milestone was achieved in the development of internet hospitals. The State Council issued a programmatic policy that nationally recognized internet hospitals for the first time. Subsequently, the National Health Commission and the National Administration of Traditional Chinese Medicine issued the “Internet Medical Consultation Administrative Measures (Trial),” “Telemedicine Services Administrative Standards (Trial),” and “Internet Hospital Administrative Measures (Trial)” policies. These polices defined the access, practice, and supervision rules and measures of internet hospitals. In 2019, the National People's Congress issued the Law of the People's Republic of China on Pharmaceutical Administration, which clearly supported the development of “Internet + Health Care” hospitals and allowed the web-based sale of prescription drugs at the legislative level. The National Health Security Administration issued guidance on “Internet +” medical service prices and medical insurance payment policies in an attempt to support the medical insurance coverage of internet hospitals.

**Figure 2:**
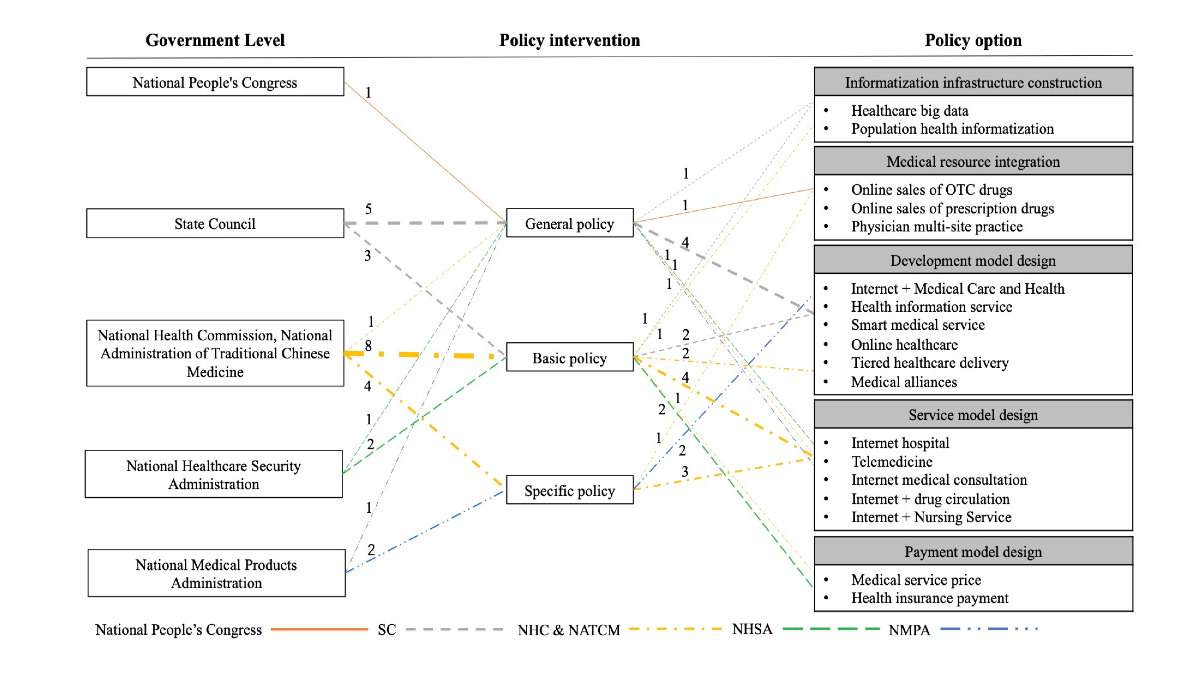
The evolution of policy options for internet hospitals in China. The numbers represent the number of policy documents. NATCM: National Administration of Traditional Chinese Medicine; NHC: National Health Commission; NHSA: National Healthcare Security Administration; NMPA: National Medical Products Administration; OTC: over-the-counter; SC: State Council.

In general, with regard to policy focus, phase 1 emphasized informatization infrastructure construction, medical resource integration, and development model design; phase 2 prioritized the transformation of resources into effective services through systemic service model design and payment model design. A detailed analysis of policy makers, policy levels, and policy options are summarized in Figure 2.

### Development Trends of Internet Hospitals in China

**Figure 3:**
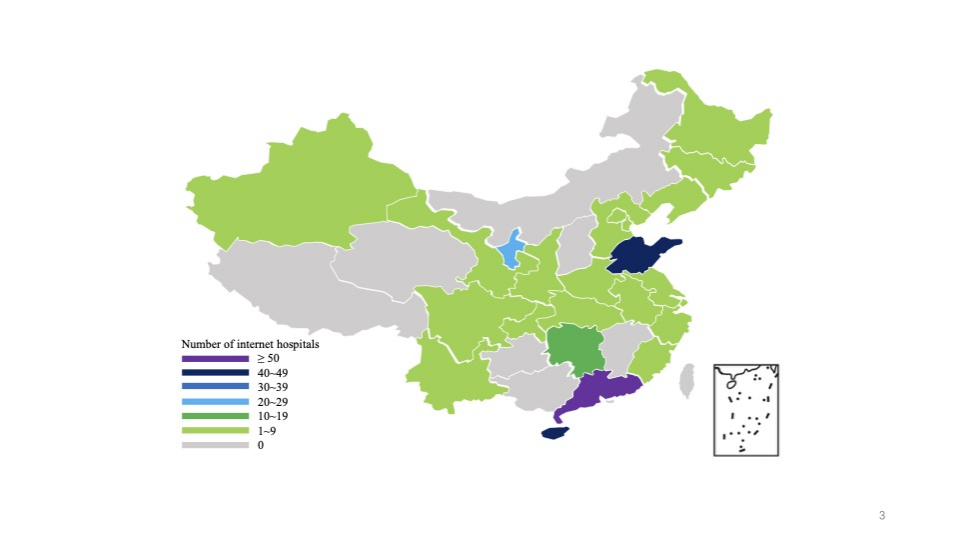
The geographical distribution of internet hospitals in China.

Until April 15, 2020, a total of 268 internet hospitals received official licenses from the government that were distributed in 24 provinces (municipalities) in China (Figure 3). Guangdong Province, Shandong Province, and Hainan Province approved more than 40 internet hospitals and thus played leading roles in the development of internet hospitals.

**Figure 4:**
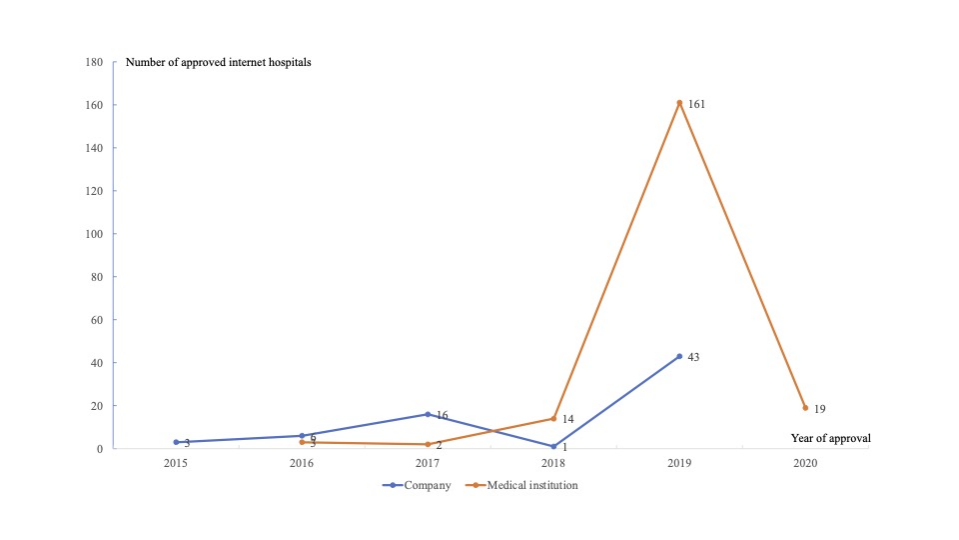
Approval of internet hospitals in China. No internet hospitals that were founded by companies were approved between January 1, 2020, and April 15, 2020.

As shown in Figure 4, the establishment of internet hospitals increased quickly since 2019. This was the result of the new policy support released in 2018. In 2019, a total of 204 internet hospitals were approved (including 161 internet hospitals founded by medical institutions and 43 internet hospitals founded by companies), accounting for 76.1% (204/268) of internet hospitals in China.

**Figure 5:**
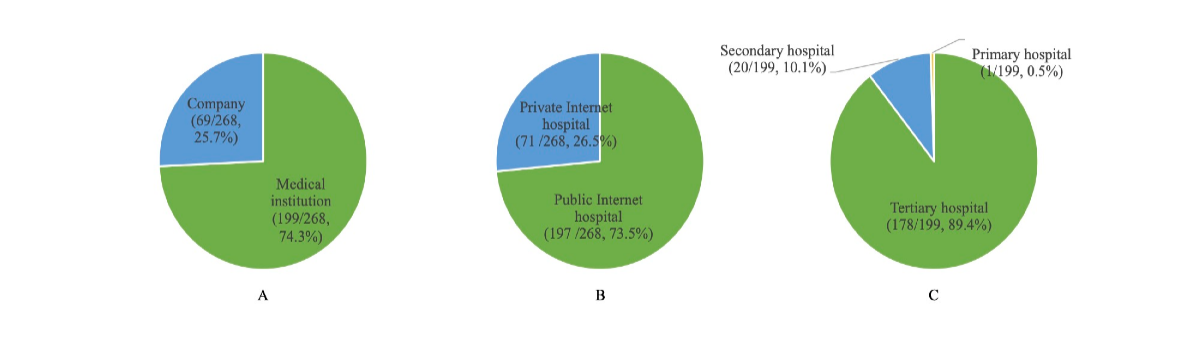
Proportions of internet hospital types. A: The proportions of internet hospitals developed by medical institutions and companies. B: The proportions of public internet hospitals and private internet hospitals. C: The proportions of internet hospitals established by different levels of medical institutions.

Of the 268 approved internet hospitals, 199 (74.3%) internet hospitals were established by medical institutions, which is much higher than the number of internet hospitals founded by companies such as internet companies and risk investment companies (Figure 5). This implies that medial institutions have more resources and the capacity to develop internet hospitals. With regard to the ownership of internet hospitals, there were 197 public internet hospitals that were mainly established by medical institutions and 71 private internet hospitals (Figure 5).

In total, 199 internet hospitals were founded by medical institutions; the number of internet hospitals established by tertiary hospitals, secondary hospitals, and primary hospitals was 178 (89.4%), 20 (10.1%), and 1 (0.5%), respectively (Figure 5). These data show that tertiary hospitals, which have the absolute advantage in terms of medical resources, have more motivations and the ability to establish internet hospitals. Furthermore, 36.4% (75/206) of tertiary hospitals in Guangdong Province founded their internet hospitals, 23.1% (3/13) of tertiary hospitals in Ningxia Province founded their internet hospitals, and 22.7% (41/181) of tertiary hospitals in Shandong Province founded their internet hospitals (Table 2).

**Table 2:** Development of internet hospitals established by different levels of medical institutions.

Location	Tertiary hospitals (N=178), n	Secondary hospitals (N=20), n	Primary hospitals (N=1), n	Internet hospitals in tertiary hospitals^a^, n (%)
Guangdong	75	6	0	75 (36.4)
Shandong	41	7	0	41 (22.7)
Hunan	10	0	0	10 (12.7)
Jiangsu	8	0	0	8 (5)
Shanghai	4	1	0	4 (8.5)
Yunnan	2	3	0	2 (2.9)
Sichuan	5	0	0	5 (2.5)
Fujian	5	0	0	5 (6.5)
Gansu	5	0	0	5 (13.5)
Auhui	5	0	0	5 (7.4)
Zhejiang	3	0	1	3 (2.2)
Liaoning	3	0	0	3 (2.2)
Ningxia	3	0	0	3 (23.1)
Heilongjiang	2	0	0	2 (2.1)
Tianjin	2	0	0	2 (4.7)
Shanxi	1	1	0	1 (1.6)
Xinjiang	0	1	0	0 (0)
Hebei	1	0	0	1 (1.4)
Beijing	1	0	0	1 (1)
Hubei	1	0	0	1 (0.8)
Henan	1	0	0	1 (4.6)
Jinlin	0	1	0	0 (0)

^a^The total proportion of internet hospitals in tertiary hospitals was 8.8% (178/2015).

### Service Innovations of Internet Hospitals in China

Unlike traditional medical institutions, internet hospitals in China have tried to implement service innovations, which can be categorized based on the following three aspects: the target patients of internet hospitals, value offerings of internet hospitals, and the services provided by internet hospitals.

#### Target Patients of Internet Hospitals

With regard to the target patients of internet hospitals, there are two main types of patients—patients with common diseases or chronic diseases and patients in remote and rural areas.

#### Patients With Common Diseases or Chronic Diseases

At present, internet hospitals are not allowed to provide medical services to first-diagnosis patients due to certain policies. Internet hospitals were mainly designed to provide medical services to countercheck patients with common diseases or chronic diseases. An interviewee said:

At present, the Internet hospital is still in the exploratory development stage. For the sake of people's life, health and safety, the first step is to open only to the patients with common diseases or chronic diseases. When the conditions are mature, we will gradually open to the first-diagnosis patients.

Interviewee #2, policy maker at the National Health Commission

#### Patients in Remote and Rural Areas

Due to objective conditions such as economic underdevelopment and a lack of medical resources, problems such as an insufficient number of primary physicians and the unmet health care needs of primary patients in remote and rural areas are very prominent. Internet hospitals were thus positioned to meet the unmet medical service needs of patients in remote and rural areas. This was described by two interviewees, as follows:

For example, some stroke patients from rural areas do not know how to recover at home after discharge. But the village doctors there are unable to provide professional advice either. In such kind of situation, it needs specialist doctor (of Internet hospital) to tell the patients what to do and how to do.

Interviewee #5, manager of an internet company

Every village doctor in Guangdong Province manages more than 1,000 patients on average, even more than 5,000 patients. They can not meet the needs of health screening, health follow-up, and clinical instruction of so many patients.

Interviewee #15, doctor at a public hospital

#### The Key Values of Internet Hospitals

Unlike the traditional medical institutions in China, internet hospitals might offer the following three distinctive values: saving patients’ and their families’ time, providing more convenient access to high-quality medical resources, and expanding from treatment-oriented services to complete health management.

#### Saving Patients’ and Their Families’ Time

One of the main problems that result in poor medical experiences in China is the time cost of medical services at traditional hospitals, including long registration times, long waiting times, short medical consultation times, and long times for taking medicine. With the help of internet hospitals, these problems could be greatly improved. This was expressed by two interviewees, as follows:

80% of the patients in Southern Hospital are non-local. It is very troublesome for countercheck patients every time. For example, a patient with asthma in Hunan Province needs to spend three days per month to take a high-speed train to come to the hospital for medical consultation and medicine. The emergence of Internet hospital can solve this problem well. He only needs to communicate with the doctor through pictures and texts or video, and the doctor can send the medicine by express to him after diagnosis, which can save a lot of time for patient.

Interviewee #29, management staff member of a public hospital

The most obvious advantage of Internet hospital is the convenience of registration, waiting, medical consultation and medicine taken, so as to avoid wasting time and energy.

Interviewee #32, policy maker at the City Health Commission

#### More Convenient Access to High-Quality Medical Resources

Internet hospitals overcame geographical restrictions to provide medical services to grassroots patients directly. In theory, with the help of internet hospitals, patients can obtain medical services from any hospital, including exceptionally scarce, high-quality medical resources at high-level medical institutions. This was described by two interviewees, as follows:

Internet hospital is mainly to solve the medical problems and alleviate the low competence challenge of doctors in primary hospitals.

Interviewee #5, manager of an internet company

Internet hospital has two core functions: one is to solve the shortage of human resources in primary hospitals; the other is to solve the uneven distribution of medical resources. For example, in Guangdong Province, 80% of medical resources are concentrated in Guangzhou City, but 90% of patients are at other cities.

Interviewee #15, doctor of a public hospital

#### Service Expansion From Treatment-Oriented Services to Complete Health Management Services

Unlike the traditional medical institutions that focused primarily on medical treatment, internet hospitals were expected to provide complete health management services for patients and ordinary persons. This was explained by two interviewees, as follows:

Large hospitals only solve the short-term problem of patients' acute attack. But patients' later rehabilitation is more important, including patients' health recording and health education, which is the biggest problem of medical system. We should consider how to make good use of Internet hospital for health management at the grass-roots level.

Interviewee #15, doctor of a public hospital

The development of Internet hospitals should insist on people-centered and expand medical services from treatment-centered to health-centered.

Interviewee #2, policy maker at the National Health Commission

#### Services Provided by Internet Hospitals

According to the service process, the main services provided by internet hospitals can be categorized as web-based registration services, web-based medical consultation services, web-based drug supply services, and web-based payment services.

#### Web-Based Registration Service

Internet hospitals provide web-based registration services for both outpatients of physical medical institutions and patients attending web-based medical consultations. The web-based outpatient registration of physical medical institutions covered all medical departments. Comparatively, web-based registration for web-based medical consultations was only open to patients with common diseases or chronic diseases. The was described by an interviewee, as follows:

In the past, online registration was only used for outpatients of physical medical institution, for patient convenience. Now it (online registration service) has become the entrance of Internet hospitals, having the function of triage patients.

Interviewee #40, management staff member of a public hospital

#### Web-Based Medical Consultation Service

According to different service models, consultation services can be divided into three types—image-text medical consultations, telephone medical consultations, and video medical consultations. These are different in terms of service objects, service content, and prescriptions.

With regard to image-text medical consultations, patients need to fill in the application form for medical consultations, which includes patients’ health-related information (eg, condition description, past medical history, allergies, family genetic diseases, marriage, and birth status, and personal habits). During this time, patients can provide relevant health-related information by sending images. For counterchecked patients, physicians could make web-based diagnoses and order a prescription after reviewing specific health-related information. For first-diagnosis patients, physicians could only provide health consulting services that did not involve disease diagnosis and prescription.

With regard to telephone medical consultations, patients first need to fill in the application form for medical consultations first before scheduling a time with the doctor for telephone medical consultation. However, doctors can only provide health consultations; they cannot provide disease diagnoses and prescriptions.

Video medical consultations could be used for counterchecking patients. After completing the application form, video medical consultations are carried out at the time that was agreed upon by both the patients and the physicians. The physician can then make a web-based diagnosis and order a prescription after reviewing specific health-related information. An interviewee presented his ideas about the three types of web-based consultation services, as follows:

Image-text medical consultation, telephone medical consultation, and video medical consultation are three common service models at present. In fact, most patients choose telephone consultation because it is convenient and not as troublesome as text description. However, from the perspective of hospital or doctor, they prefer image-text and video consultation, it’s more objective and accurate, and is easy to keep records.

Interviewee #34, management staff member of a public hospital

#### Web-Based Drug Supply Service

After making a web-based disease diagnosis and offering a prescription, internet hospitals can provide web-based drug services. Internet hospitals can either send drugs to patients directly or transfer prescriptions via web-based platforms to designated community pharmacies that were near patients. Two interviewees described this service, as follows:

At present, online prescriptions only flow to the pharmacy of the hospital,.... Patients can go to get the drugs directly or send drugs by express to their home.

Interviewee #29, management staff member of a public hospital

For example, in Wuzhou City, the city platform of prescription circulation has been established. Community pharmacies joining the platform can accept online prescription orders. Patients can pick up drugs at the community pharmacy around their home or receive drugs by express delivery.

Interviewee #51, academic researcher at a university

#### Web-Based Payment Service

Internet hospitals can provide web-based payment services for expenses related to registration, medical consultations, and drugs. Due to the differences in the progress of medical insurance reform in different regions, the web-based settlement of medical insurance had only been realized in a few regions of China. However, patients need to pay their expenses if there was no web-based medical insurance settlement. This was explained by two interviewees, as follows:

Medical insurance is often the last one that gets involved. As the payer, we must be cautious. Moreover, the medical insurance policy of Internet hospital has not been fully developed in the country, mainly for pilots. Only a few provinces, such as Shandong Province and Fujian Province, have issued specific policies, such as reimbursement items and price.

Interviewee #22, policy maker at the City Health Commission

If the Internet hospital is not connected to medical insurance, all the expenses of patients in the Internet hospital need to be paid by themselves and cannot be reimbursed.

Interviewee #17, internet hospital manager of an internet company

## Discussion

### Principal Findings

This study reported a detailed overview of the policy interventions, development trends, and service innovations of internet hospitals in China. The understanding of these internet hospital aspects is lacking in existing literature. Our findings contribute to the understanding of policy development and implementation in the internet hospital sector. Based on our findings, there are some points that are worthy of further discussion.

First, after collating and summarizing new policies for internet hospitals, it became apparent that the multiple policy interventions introduced in the internet hospital sector over the past 15 years reflected policy makers’ determination in firmly supporting the development of internet hospitals. Moreover, these continuous policy interventions gave stakeholders the confidence to found internet hospitals and integrate them into the entirety of the health system. This is particularly important for resource-limited countries such as China, where there is a rising demand for health care but an uneven distribution of medical resources [44]. Although the Chinese government has been promoting the development of a tiered health care delivery system that is anchored in primary health care, the actual implementation of this system deviated substantially from the ideal model [45,46]. Therefore, based on the rapid development of internet hospitals in China, the medical care and health services of medical institutions at all levels can be coordinated and integrated, which can lead to the improvement of medical alliances and tiered health care delivery [47]. It would be beneficial to solve the problem of the uneven distribution of medical resources, innovate health care, and improve the efficiency of medical services.

Based on the evolution of policy interventions, internet hospitals feature the concept of “experimentation precedes popularization” [48]. The first step in achieving these goals was to identify potential problems by piloting web-based sales of over-the-counter drugs and telemedicine services. In the second step, informatization infrastructure construction, medical resource integration, and development model design were carried out based on the pilot tests. In the final step, the service model and payment model were introduced to strengthen the development of internet hospitals. All of these processes show that the Chinese government, which is cautious about the development of internet hospitals because they are a type of unknown medical institution, has taken a step-by-step approach to design and implement policies related to these five processes. These policy designs provide valuable references to other countries’ policy development processes for internet hospitals.

Second, this study showed that public tertiary hospitals played a more leading role in establishing internet hospitals compared to the role of companies, such as large internet companies. This is consistent with previous research findings [49]. Since medical resources are mainly found at tertiary hospitals, they have distinct advantages in terms of medical equipment, finances, physicians, and patient sources [50]. Furthermore, public tertiary hospitals employ most of the high-level physicians and own most of the medical facilities in China [51]. Therefore, most of the existing internet hospitals were founded by public tertiary hospitals. Although internet companies have inherent advantages in terms of information technology and their ability to operate the internet, which are lacking in most traditional hospitals, they still cannot directly compete with public tertiary hospitals in China. In practice, internet companies have to cooperate with medical institutions to enter the sector of internet hospitals [52]. All of these findings indicate that high-quality medical resources like physicians and medical equipment are the key factors that shape the development of internet hospitals.

Third, the services provided by internet hospitals are particularly valuable to patients with common diseases or chronic diseases and patients in remote and rural areas. First-diagnosis patients are still unable to access the services provided by internet hospitals. In theory, the most common barriers that stop patients from obtaining satisfactory medical services are cost, access, skill, and time [53]. High-quality medical resources are mainly found in public tertiary hospitals [54]. However, due to financial and professional reasons, skilled physicians are unwilling to work in communities or remote and rural areas [55]. Additionally, many primary patients are reluctant to go to primary hospitals due to a lack of confidence in health professionals’ skills and the quality of health care provided [56]. With the operation model that is currently in place, internet hospitals can reinforce the interactions between physicians and patients without the challenges of geographical limitations; increase the accessibility of high-quality medical resources in remote and rural areas; and dramatically reduce the indirect costs of medical care for patients, especially the expenses and time associated with travel [57]. As a result, regardless of the service type or service content, internet hospitals can at least play a role that is complementary to that of physical hospitals. Furthermore, due to innovations such as web-based registration services, web-based medical consultations, web-based drug supply and support services, and web-based payment services, internet hospitals can provide patients with more convenient methods for accessing high-quality medical resources and save patients’ and their families’ time. However, internet hospitals do not have direct cost and skill advantages.

In general, this study found that the government has provided a lot of space for the development of internet hospitals in China, but the value of internet hospital services has not been fully realized in operation. Thus, we propose the following suggestions for the further development of internet hospitals. First, although general policy interventions regarding medical insurance for internet hospital services have been issued, there are no specific implementation instructions at the national level. In particular, there is a need to introduce specific medical insurance measures at the national level as implementation guidance for local governments. Second, to motivate physicians at public tertiary hospitals to support the platform provided by internet hospitals, policies for encouraging physicians at public tertiary hospitals to join internet hospitals and providing reasonable incentives are needed. Third, policies for encouraging the sharing of medical facilities and equipment among internet hospitals and physical medial institutions are necessary to further the service innovations of internet hospitals.

### Limitations

To the best of our knowledge, this is the first study that has collated and evaluated multiple policy interventions that were designed to influence internet hospital development in China. There are several research limitations that can be addressed in future studies. First, in this study, we did not collect data from patients. Further studies can focus on patients’ realistic experiences with internet hospitals. Second, public hospital and internet companies have different strategies for developing internet hospitals, and these require further comparison and analysis.

### Conclusions

Policy interventions regarding informatization infrastructure construction, medical resource integration, development model design, service model design, and payment model design have significantly promoted the development of internet hospitals in China. Further, public tertiary hospitals play a more leading role in founding internet hospitals compared to the role of internet companies. The service innovations of internet hospitals need to be further advanced with the support of corresponding policy interventions.

## Appendix

Multimedia Appendix 1 Details of the 25 policy documents.
