# Supplementary material for: Policy Interventions, Development Trends, and Service Innovations of Internet Hospitals in China: Documentary Analysis and Qualitative Interview Study
Source: J Med Internet Res. 2021 Jul 20;23(7):e22330. doi: 10.2196/22330 (PMC8335616; doi:10.2196/22330)
Supplement: Multimedia Appendix 1 [file jmir_v23i7e22330_app1.docx]

# *Multimedia Appendix 1*

**Details of the 25 policy documents.**

| **Policy** | **Initiating bodies** | **Year introduced** | **Type of policy** | **Objective** | **Process to achieve objective** | **Policy option** | **Resource sources** |
| --- | --- | --- | --- | --- | --- | --- | --- |
| Provisional Regulation on the Approval of Internet Drug Business | State Food and Drug Administration | September 2005 | Specific policy | To explore the online sales of over-the-counter drugs | To explore the online sales of over-the-counter drugs | The online sales of over-the-counter drugs | <http://www.gov.cn/govweb/fwxx/bw/spypjgj/content_505677.htm> |
| Notice to strengthen the administration of Internet drug sales | China Food and Drug Administration | October 2013 | Specific policy | To regulate the online sales of over-the-counter drugs in pharmacies | To regulate the online sales of over-the-counter drugs in pharmacies | The online sales of over-the-counter drugs | http://www.gov.cn/gzdt/2013-10/29/content_2517631.htm |
| Opinions of National Health and Family Planning Commission on promoting telemedicine service in medical institutions | National Health and Family Planning Commission | August 2014 | Basic policy | To develop telemedicine service | Telemedicine service is allowed for the first time | Telemedicine | http://www.nhc.gov.cn/yzygj/s3593g/201408/f7cbfe331e78410fb43d9b4c61c4e4bd.shtml |
| Planning of the General Office of the State Council on the national medical and health service system (2015-2020) | General Office of the State Council | March 2015 | General policy | To clear development plan of the national medical and health service system | To clear promote the development of mobile Internet and telemedicine services. To promote health information service and smart medical service benefiting the whole people by information technology. | Health information service; Smart medical service; Electronic health archive; Electronic medical record; Population health information platform; Telemedicine; Resident health card | http://www.gov.cn/zhengce/content/2015-03/30/content_9560.htm |
| Guidance of the State Council on promoting "Internet +" action | State Council | July 2015 | Basic policy | To define the direction of "Internet +" in the medical field | To promote the new model of online health care. To support the development of medical information sharing service platform by the third-party institutions. To provide convenient services of online appointment diagnosis and treatment and drug distribution by mobile Internet. To provide telemedicine services to the grass-roots. To improve the prevention and control ability of major diseases and public health emergencies by the Internet and big data. To explore the development of electronic prescription. | Medical and health information platform; Mobile Internet medical; Telemedicine; Public health prevention and control; Electronic prescription | http://www.gov.cn/zhengce/content/2015-07/04/content_10002.htm |
| Guidance of the General Office of the State Council on promoting tiered healthcare delivery system | General Office of the State Council | September 2015 | Basic policy | To promote the development of tiered healthcare delivery by information technology and big data | To develop Internet-based medical and health services.To establish a regional medical and health information platform. To realize the sharing of electronic health records and electronic medical records. To improve the ability of telemedicine services. | Medical and health information platform; Electronic health archive; Electronic medical record; Telemedicine | http://www.gov.cn/zhengce/content/2015-09/11/content_10158.htm |
| Guidance of the State Council on promoting and standardizing the application and development of healthcare big data | General Office of the State Council | June 2016 | Basic policy | To define the development of healthcare big data | To clear the development goals of healthcare big data, including the overall goals and specific goals, and emphasize its application and innovation. | Healthcare big data; Population health information platform; Application platform of national drug bidding and purchasing business; Medical and health information platform; Electromic health archive; Resident health card | http://www.gov.cn/zhengce/content/2016-06/24/content_5085091.htm |
| Healthy China 2030 Plan | The CPC Central Committee, the State Council | October 2016 | General policy | To integrate "Internet + Medical Care and Health" into the national strategic planning | The development of health care industry has been upgraded to the national level strategy for the first time, and clearly supported the development of "Internet + Medical Care and Health". | Population health information platform; Internet healthcare; Telemedicine; Electromic health archive; Healthcare big data | http://www.gov.cn/xinwen/2016-10/25/content_5124174.htm |
| Notice of the State Council on the 13th five-year plan for healthcare | State Council | January 2017 | General policy | To define the development strategy of population health informatization | Implementation of population health informatization construction project, including to promote the exchange and sharing of population health information platform and the rapid and orderly development of new business forms of healthcare information. | Population health information platform; Electromic health archive; Electronic medical record; Resident health card; Network security; Information security; Telemedicine; | http://www.gov.cn/zhengce/content/2017-01/10/content_5158488.htm |
| Notice of National Health and Family Planning commission on the national population health informatization development in the 13th five-year | National Health and Family Planning Commission | February 2017 | Basic policy | To define the development measures of population health informatization | Three major tasks and five key projects for the development of population health informatization have been formulated. | Population health information; Healthcare big data | http://www.nhc.gov.cn/guihuaxxs/s10741/201702/ef9ba6fbe2ef46a49c333de32275074f.shtml |
| Guidance of the General Office of the State Council on promoting the construction and development of medical alliances | General Office of the State Council | April 2017 | General policy | To promote the development of medical alliances by information technology and big data | To develop various forms of medical alliances by information technology and big data, and promote tiered healthcare delivery. | Population health information platform; Electromic health archive; Electronic medical record; Information interconnection | http://www.gov.cn/zhengce/content/2017-04/26/content_5189071.htm |
| Regulatory Practices for the Registration of Physician Practices | National Health and Family Planning Commission | April 2017 | Specific policy | To standardize the management of physician Practices | To allow physician multi-site practice. | Physician multi-site practice | http://www.nhc.gov.cn/fzs/s3576/201808/a359f40edf5e43a79d0174437fdca02f.shtml |
| Action plan for further improvement of medical services (2018-2020) | National Health and Family Planning Commission, National Administration of Traditional Chinese Medicine | January 2018 | Basic policy | To define development strategies of innovative medical service mode | Based on the Internet and big data, five reform measures of hospital work system and 10 innovative medical service modes were defined. | Innovative medical service model; Smart hospital | http://www.gov.cn/gongbao/content/2018/content_5299607.htm |
| State Council's opinion about promoting the development of "Internet + Medical Care and Health" | General Office of the State Council | April 2018 | General policy | Programmatic policy for developing "Internet + Medical Care and Health" | To define the development direction, service system, supporting system, supervision system and safeguard system of "Internet + Medical care and Health". | Internet + Medical Care and Health; Medical services; Public health services; Family physician contract services; Drug supply and support services; Healthcare insurance settlement services; Medical education and science popularization services; AI application services | http://www.gov.cn/zhengce/content/2018-04/28/content_5286645.htm |
| Notice to further develop "Internet + Medical Care and Health" to benefit the people | National Health Commission, National Administration of Traditional Chinese Medicine | July 2018 | Basic policy | To promote the development of specific measures based on "Internet + Medical Care and Health" | To develop "Internet + Medical Care and Health" services from 10 aspects: medical treatment, settlement payment, patient medication, public health, family doctor, telemedicine, health information, emergency treatment, government affairs sharing and inspection and inspection. | Smart hospital; Online booking medical consultation platform; Internet hospital; Online payment; Smart pharmacy; Health management; Telemedicine; Government affairs sharing | http://www.gov.cn/zhengce/zhengceku/2018-12/31/content_5435186.htm |
| Internet Medical Consultation Administrative Measures (Trial) | National Health Commission, National Administration of Traditional Chinese Medicine | Setpember 2018 | Specific policy | To formulate the administrative measures of Internet medical consultation | To define the access, practice rules and supervision measures of Internet medical consultation. | Administrative measures | http://www.nhc.gov.cn/yzygj/s3594q/201809/c6c9dab0b00c4902a5e0561bbf0581f1.shtml |
| Internet Hospital Administrative Measures (Trial) | National Health Commission, National Administration of Traditional Chinese Medicine | Setpember 2018 | Specific policy | To formulate the administrative measures of Internet hospital. | To define the access, practice rules and supervision measures of Internet hospital. | Administrative measures | http://www.nhc.gov.cn/yzygj/s3594q/201809/c6c9dab0b00c4902a5e0561bbf0581f1.shtml |
| Telemedicine Services Administrative Standards (Trial) | National Health Commission, National Administration of Traditional Chinese Medicine | Setpember 2018 | Specific policy | To formulate the administrative standards of telemedicine services. | To define the management scope, basic conditions, service process, management requirements and supervision measures of telemedicine services. | Administrative standards | http://www.nhc.gov.cn/yzygj/s3594q/201809/c6c9dab0b00c4902a5e0561bbf0581f1.shtml |
| Notice of National Health Commission to carry out pilot work of "Internet + Nursing Service" | National Health Commission | January 2019 | Basic policy | To explore the development of "Internet + Nursing Service". | To explore the development of "Internet + Nursing Service", including the development goals, the pilot services, the pilot areas and the pilot time. | Internet + nursing service | http://www.nhc.gov.cn/yzygj/s7657g/201902/bf0b25379ddb48949e7e21edae2a02da.shtml |
| Law of the People's Republic of China on Pharmaceutical Administration | National People's Congress | August 2019 | Legislation | To allow the online sales of prescription drugs from the legislative level. | To support the development of "Internet + Medical Care and Health". To allow the online sale of prescription drugs from the legislative level. | The online sales of prescription drugs | http://www.gov.cn/xinwen/2019-08/26/content_5424780.htm |
| Guidance of the National Healthcare Security Administration on improving the "Internet +" medical service price and healthcare insurance payment policy | National Healthcare Security Administration | August 2019 | Basic policy | To clear the policy guidance for the healthcare insurance settlement of "Internet +" medical services. | The "Internet +" medical service that meets certain conditions can be included in the coverage of healthcare insurance reimbursement. To implement online and offline integrated management. | Medical service price; Healthcare insurance payment | http://www.nhsa.gov.cn/art/2019/8/30/art_37_1707.html |
| Notion of multi sectors of action plan on promoting high quality development of health industry (2019-2022) | National Development and Reform Commission, Ministry of Education, Ministry of Science and Technology, Ministry of Industry and Information Technology, Ministry of Civil Affairs, Minitry of Finacne, Ministry of Human Resources and Social Security, Ministry of Natural Resources, Ministry of Ecology and Environment, Ministry of Housing and Urban-Rural Development, Ministry of Commerce, Ministry of Culture and Tourism, National Health commission, The People's Bank of China, State Taxation Administration, State Administration for Market Regulation, General Administration of Sport of China, China Banking and Insurance Regulatory Commission, National Healthcare Security Administration, National Administration of Traditional Chinese Medicine, National Medical Products Administration | August 2019 | General policy | To implement the upgrade project for "Internet + Medical Care and Health". | To develop the national health information platform. To apply the healthcare big data. To accelerate the development of "Internet + Medical". To develop "Internet + drug circulation". | National health information platform; Healthcare big data; Internet + Medical; Internet + drug circulation | http://www.gov.cn/xinwen/2019-09/30/content_5435160.htm |
| Notice on strengthening information to support the prevention and control work of the pneumonia caused by novel coronavirus infection | National Health Commission | February 2020 | Basic policy | To strengthen informatization to support epidemic prevention and control | To strengthen data analysis and application. To develop telemedicine services. To improve Internet medical consultation. To improve "Internet +" government affairs services. | Internet hospiatl; Telemedicine; Internet medical consultation | http://www.gov.cn/zhengce/zhengceku/2020-02/05/content_5474692.htm |
| Notice on Promoting Internet medical consultation services in epidemic prevention and control | National Health Commission | February 2020 | Basic policy | To strengthen Internet medical consultation to support epidemic prevention and control | To promote the prevention, control, education and science popularization of the epidemic by the Internet medical consultation service platforms and Internet hospitals, | Internet medical consultation service platform; Internet hospital; Medical education and science popularization services | http://www.nhc.gov.cn/yzygj/s7653p/202002/ec5e345814e744398c2adef17b657fb8.shtml |
| Guidance of advancing the "Internet +" healthcare insurance service in Novel coronavirus pneumonia prevention and control period | National Healthcare Security Administration, National Health Commission | March 2020 | Basic policy | To strengthen "Internet +" healthcare insurance service to support epidemic prevention and control | To define the scope of healthcare insurance payment. To encourage continuous prescriptions. To allow online payment and direct settlement. | Continuous prescription for chronic diseases; Online payment; Direct settlement | http://www.nhsa.gov.cn/art/2020/3/2/art_37_2750.html |
